# New tools for characterizing early brown stem rot disease resistance signaling in soybean

**DOI:** 10.1002/tpg2.20037

**Published:** 2020-09-14

**Authors:** Chantal E. McCabe, Michelle A. Graham

**Affiliations:** ^1^ USDA–ARS Corn Insects and Crop Genetics Research Unit Ames IA 50011‐1010 USA; ^2^ Department of Agronomy Iowa State University Ames IA 50011‐1010 USA

## Abstract

Brown stem rot (BSR) reduces soybean [*Glycine max* (L.) Merr.] yield by up to 38%. The BSR causal agent is *Phialophora gregata* f. sp. sojae, a slow‐growing, necrotrophic fungus whose life cycle includes latent and pathogenic phases, each lasting several weeks. Brown stem rot foliar symptoms are often misdiagnosed as other soybean diseases or nutrient stress, making BSR resistance especially difficult to phenotype. To shed light on the genes and networks contributing to *P. gregata* resistance, we conducted RNA sequencing (RNA‐seq) of a resistant genotype (PI 437970, *Rbs3*). Leaf, stem, and root tissues were collected 12, 24, and 36 h after stab inoculation with *P. gregata*, or mock infection, in the plant stem. By using multiple tissues and time points, we could see that leaves, stems, and roots use the same defense pathways. Our analyses suggest that *P. gregata* induces a biphasic defense response, with pathogen‐associated molecular pattern (PAMP) triggered immunity observed in leaves at 12 and 24 h after infection (HAI) and effector triggered immunity detected at 36 h after infection in the stems. Gene networks associated with defense, photosynthesis, nutrient homeostasis, DNA replication, and growth are the hallmarks of resistance to *P. gregata*. While *P. gregata* is a slow‐growing pathogen, our results demonstrate that pathogen recognition occurs hours after infection. By exploiting the genes and networks described here, we will be able to develop novel diagnostic tools to facilitate breeding and screening for BSR resistance.

**Abbreviations**
BSR: brown stem rot
DAI: days after infection
DE: differentially expressed
DEG: differentially expressed gene
ETI: effector‐triggered immunity
FDR: false discovery rate
GO: gene ontology
HAI: hours after infection
JA: jasmonic acid
MAPK: mitogen‐activated protein kinase
NBS‐LRR: nucleotide‐binding site–leucine‐rich repeat
PAMP: pathogen‐associated molecular pattern
PTI: PAMP‐triggered immunity
RNA‐seq: RNA sequencing
SA: salicylic acid
SMV: soybean mosaic virus
TF: transcription factor
TFF: transcription factor family
WAI: weeks after infection.

## 1 INTRODUCTION

The soil‐borne fungus *P. gregata* (Allington & Chamberlain, 1948) (syn. *Cadophora gregata*) (Harrington & McNew, 2003) is responsible for the soybean disease BSR. First identified in the United States in 1944 (Allington & Chamberlain, 1948), BSR has rapidly spread throughout all major soybean producing regions of the United States and Canada (Malvick & Grunden, 2008). Brown stem rot is a major soybean disease, with yield losses up to 38% reported (Bachman & Nickell, 2000). Two types of *P. gregata* have been identified: Type A and Type B (Gray, 1972; Harrington, Steimel, Workneh, & Yang, 2003). Infection with either Type A or Type B of *P. gregata* results in internal stem symptoms appearing as browning of the pith and vascular tissue. In addition, leaves of plants infected with *P. gregata* Type A produce foliar symptoms, whereas plants infected with *P. gregata* Type B appear healthy and normal. The foliar symptoms produced by Type A emerge as leaf necrosis and chlorosis. *Phialophora gregata* is a latent necrotroph, which means it can persist in plant tissues without causing the development of disease symptoms until several weeks after infection (Impullitti & Malvick, 2014). Further, BSR foliar symptoms are often misdiagnosed as other soybean diseases or nutrient stresses, making BSR especially difficult to phenotype.

Brown stem rot management options are limited to genetic resistance and long‐term crop rotation (Adee, Grau, & Oplinger, 1997). The use of BSR‐resistant cultivars has been the most effective method of managing the disease. Three resistance loci have been identified through allelism studies: *Rbs1*, *Rbs2*, and *Rbs3* (Hanson, Nickell, Gray, & Sebastian, 1988; Willmont & Nickell, 1989). All three loci have been mapped to an overlapping region on soybean chromosome 16 (28.9–36.2 Mbp) (Bachman, Tamulonis, Nickell, & Bent, 2001; Klos, Paz, Fredrick Marek, Cregan, & Shoemaker, 2000; Lewers et al., 1999). Combining fine‐mapping approaches (Rincker, Hartman, & Diers, 2016) with a genome‐wide association study (Rincker, Lipka, & Diers, 2016) suggested that the three known *Rbs* genes are actually a single gene located within a 41‐kb interval on chromosome 16. Unfortunately, these discrepancies made it challenging to identify candidate resistance genes and to integrate BSR resistance into breeding programs. In addition, the narrow genetic base of BSR resistance is of great concern as there are several reports of BSR symptoms on soybean lines containing one or more known *Rbs* genes (Bachman, Nickell, Stephens, & Nickell, 1997; Hanson et al., 1988; Nelson et al., 1989).

New tools are needed to identify and understand the genes and gene networks underlying BSR resistance and ultimately develop elite soybean lines with enhanced BSR resistance. Several groups have leveraged candidate genes identified through gene expression studies with virus‐induced gene silencing to identify and characterize soybean genes required for defense against fungal, bacterial, and viral pathogens (Liu & Whitham, 2013; Pandey et al., 2011; Selote, Robin, & Kachroo, 2013; Wang et al., 2014; Zhang, Grosic, Whitham, & Hill, 2012). Therefore, we are using high‐throughput, whole‐genome expression analyses to characterize responses to *P. gregata*. Our previous study examined transcriptional changes in leaves, stems, and roots 7 d after infection (DAI) with *P. gregata* in a resistant and susceptible genotype (McCabe, Cianzio, O'Rourke, & Graham, 2018). While that analysis identified thousands of genes differentially expressed (DE) in response to *P. gregata* infection, the largest response was observed in leaf tissue of the resistant genotype with fewer genes DE in stems and roots. This suggested that by 7 DAI, the defense response in stems and roots had already passed. This was surprising, as resistant and susceptible plants cannot be distinguished by phenotype until 5 wk after infection (WAI).

**Core Ideas**

- *Phialophora gregata* induces a biphasic defense response in soybean.
- *P. gregata* recognition occurs within hours of infection.
- Photosynthesis and growth are repressed to provide defense resources.
- Jasmonic‐acid‐mediated signaling is a major resistance component.
- Resistance signaling is observed in leaves, stems, and roots.

Therefore, the objectives of this study were to use RNA‐seq to characterize the genes and gene networks responding to *P. gregata* infection in the same resistant soybean genotype 12, 24, and 36 HAI. These time points were selected based on previous reports on the fungal biology, our previous study (McCabe et al., 2018), and a careful review of the soybean disease resistance literature. Allington and Chamberlain (1948) reported germination of conidia 24 h after treatment in distilled water or soybean‐stem juice. This suggests the fungus could also penetrate plant tissues at 24 h, eventually leading to disease symptoms. Tabor, Tylka, and Bronson (2007) assessed stem colonization of a resistant and susceptible soybean line at 1 and 24 h after puncture inoculation with *P. gregata* Type A at the base of the stem. *Phialophora gregata* could be recovered from the top of the stems in both lines, suggesting fungal spores could move readily through the plant vascular system. Repeating the experiment and evaluating at weekly intervals from 1 to 5 WAI determined that *P. gregata* advanced with the growing tips of susceptible plants but not resistant plants. Impullitti and Malvick (2014) examined the latent and pathogenic phases of *P. gregata* infection in resistant and susceptible soybean lines 1, 2, 4, 6 and 8 WAI using a root‐dip infection method. Although foliar and stem symptoms were not visible 2 WAI, Impullitti and Malvick (2014) detected *P. gregata* in resistant and susceptible stems microscopically. Review of the plant disease literature (Kim et al., 2011; Klink, Overall, Alkharouf, MacDonald, & Matthews, 2007; Lanubile, Muppirala, Severin, Marocco, & Munkvold, 2015; Li et al., 2008; Lin et al., 2014; Morales et al., 2013; Schneider et al., 2011; van de Mortel et al., 2007; Yuan et al., 2016) found time points ranging from 30 min to 72 h after infection with either bacterial or fungal pathogens or insect or nematode pests. In the majority of these cases, disease symptoms in susceptible lines were not detected for days to weeks post treatment; however, gene expression changes could be identified within hours. Remarkably, none of these studies examined the effect of pathogen or pest treatment in distal tissues. A review by Balmer and Mauch‐Mani (2013) points out the scarcity of multi tissue studies in the disease‐resistance literature and highlights potential organ‐specific defense responses identified by biochemical approaches. Using high‐throughput sequencing on three tissues and three time points allowed us to capture and characterize defense signaling throughout the plant. With this approach, we identified evidence of both the initial recognition of PAMP, leading to PAMP‐triggered immunity (PTI) and effector‐triggered immunity (ETI), leading to resistance. We identified and characterized networks of genes involved in resistance responses, confirming known resistance hubs and identifying new targets for crop improvement.

## 2 MATERIALS AND METHODS

### 2.1 Experimental design

To investigate the initial resistance response to *P. gregata*, the resistant genotype PI 437970 (Hanson et al., 1988) containing the *P. gregata* resistant gene *Rbs3* was used. Seeds were planted in a completely randomized design in a growth chamber maintained at a constant temperature of 19 ± 1.5 °C with a 16‐h photoperiod. Seedlings were grown in Metro‐Mix 900 potting soil (Sun Grow Horticulture), watered daily until saturation, and fertilized weekly with a 24‐8‐16 (P‐K‐N) fertilizer mixture.

*Phialophora gregata* Type A isolate *Oh_2‐3_* (Eathington, Nickell, & Gray, 1995; Lewers et al., 1999) cultures were grown on green bean extract medium as described by McCabe, Singh, Leandro, Cianzio, and Graham (2016). *Phialophora gregata* spores were suspended in a 1.2% water agar paste and adjusted to a final spore concentration of 2.7 × 10^7^ spores ml^−1^ (McCabe et al., 2016; Perez, Diers, Lundeen, Tabor, & Cianzio, 2010; Tabor, Tylka, Cianzio, & Bronson, 2003). Simultaneously, a spore‐free suspension consisting solely of 1.2% water agar was made, creating the mock‐infection control. Two weeks after planting, plants were stab inoculated 2 cm above the soil line with ∼20 μl of either *P. gregata* or the mock‐infection control.

Tissue samples for RNA‐seq were collected 12, 24, and 36 HAI in both the resistant‐infected and resistant‐mock treatments. At each time point, leaf, stem, and root tissue was collected from three individual plants. The first fully expanded trifoliate, the stem section between the cotyledon and the unifoliate, and the whole root were collected. All 54 samples were flash frozen in liquid nitrogen and stored at −80 °C.

### 2.2 Disease severity assay

As samples were collected before the onset of BSR symptoms; additional resistant plants and susceptible controls of Corsoy 79 (Bernard & Cremeens, 1988) were planted simultaneously with plants used for the RNA‐seq study. Five weeks after infection, 45 plants of each genotype (Corsoy 79 and PI 437970) by treatment (infected and mock) combination were phenotyped to verify expected BSR symptoms. Three standard measurements were used: foliar severity, stem severity, and stem recovery of *P. gregata* (McCabe et al., 2016). Brown stem rot foliar severity was evaluated for each plant using a scale of 1 to 7, with 1 representing most severe foliar symptoms. Stem severity was calculated as the length of brown tissue discoloration in the pith or vascular tissue compared with total plant height. To measure *P. gregata* recovery, five 1‐cm‐long sterile stem segments were plated on green bean extract agar. Two weeks later, stem segments were evaluated for the presence of *P. gregata* using a scale of 1 to 5, with 5 representing most severe infection (McCabe et al., 2016; Perez et al., 2010; Tabor et al., 2003).

### 2.3 RNA isolation and RNA sequencing

Tissue was ground with a TissueLyser (Qiagen) and RNA was extracted using a RNeasy Plant Mini Kit (Qiagen) following the manufacturer's recommended protocol. Contaminating DNA was removed with an TURBO DNA‐free kit (Ambion) and RNA was purified and concentrated using a RNeasy MiniElute Cleanup Kit (Qiagen). The 54 samples were assessed for purity and quantification using a NanoDrop ND‐1000 Spectrophotometer (Thermo Fisher Scientific) and a QIAxcel Advanced System (Qiagen). Library preparation and 100‐bp single‐end sequencing was conducted with 4 μg of total RNA using the Illumina HiSeq 2500 platform at the Iowa State University DNA facility.

### 2.4 Bioinformatic and statistical analyses

Prior to alignment, the 100‐bp reads from 54 separate libraries were trimmed to remove adaptor sequences (Scythe, https://github.com/vsbuffalo/scythe), sequencing artifacts (FASTX trimmer, http://hannonlab.cshl.edu/fastx_toolkit/), and low‐quality bases (Sickle, https://github.com/najoshi/sickle). Using TopHat version 2.0.3 (Trapnell, Pachter, & Salzberg, 2009), reads were aligned to version 2 of the Williams 82 reference genome sequence (Wm82.a2.v1; Schmutz et al., 2010). Unreliably mapped reads were removed using Samtools (Li et al., 2009) and mapping files (bamfiles) were imported into the statistical program R (R CoreTeam, 2014) using Rsamtools (Morgan & Pages, 2013). The gene feature file corresponding to the reference genome was imported using rtracklayer (Lawrence, Gentleman, & Carey, 2009). The number of reads per sample aligning to each gene were counted using GenomicAlignments (Lawrence et al., 2013) and only genes with log2 counts per million >1 in at least two replicates were used in the analysis.

For each tissue, data was normalized using the trimmed mean of mean values (Robinson & Oshlack, 2010) in the Bioconductor package edgeR (McCarthy, Chen, & Smyth, 2012; Robinson & Smyth, 2007; Robinson & Smyth, 2008; Robinson, McCarthy, & Smyth, 2010; Zhou, Lindsay, & Robinson, 2014). The R graphics program ggplot2 (Wickham, 2009) was used to generate principal component and biological coefficient of variance plots to visually compare sample replicates to ensure reproducibility (Yin et al., 2013). This analysis determined that the expression profile of eight samples were significantly different from the other samples of the same tissue and treatment. These eight samples were removed and the data was renormalized. The eight samples removed included the following: one 12‐h mock infected leaf, one 24‐h infected leaf, one 24‐h infected stem, one 24‐h infected root, one 24‐h mock infected root, one 36‐h mock infected leaf, one 36‐h infected stem, and one 36‐h infected root. For each tissue, edgeR was used to analyze differentially expressed genes (DEGs) responding to *P. gregata* infection at each time point. The DEGs were considered significant with a false discovery rate (FDR) <.05.

### 2.5 Characterization of differentially expressed genes

Differentially expressed genes were annotated using the SoyBase Genome Annotation Report page (www.soybase.org/genomeannotation). Significantly (*P*‐value < .05) overrepresented biological process gene ontology (GO) terms were identified using a Fisher's exact test (Fisher, 1960) and Bonferroni correction (Bonferroni, 1935). To cluster significant GO terms based on expression pattern, we combined all significant GO terms identified at any time point or tissue (Supplemental Table S5) into a list of 273 unique GO terms. Custom Perl scripts were used to mine Supplemental Tables S2, S3, and S4 and identify all DEGs corresponding to each of the 273 unique GO terms and determine under what tissues or time points they were significantly DE. For each GO term, the number of DEGs in each tissue and time point was determined, forming a GO term matrix. Hierarchical clustering using hclust (Murtagh & Legendre, 2014) was used to cluster GO terms in the matrix with similar gene expression patterns (Supplemental Table S6). For visualization purposes, all GO terms were expressed as a ratio of the number of DEGs in that GO term vs. the total number of genes in the reference genome associated with that GO term, allowing us to adjust for GO term size.

Transcription factors (TFs) were identified using the SoyDB TF database (Wang et al., 2010). The gene identifiers of TFs present in the database were updated to reference the new genome assembly and annotation (Wm82.a2.v1) (Schmutz et al., 2010). Significantly (*P*‐value < .05) overrepresented TFs were identified using a Fisher's exact test (Fisher, 1960) and Bonferroni correction (Bonferroni, 1935) to compare TFs at each tissue and time point to their genome representation.

Network interactions were generated using the STRING database, version 11.0 (Szklarczyk et al., 2018). The most significant *Arabidopsis thaliana* (L.) Heynh. homolog (TAIR version 10, E < 10^−6^) was identified for each significant DEG within a GO cluster. Using the *Arabidopsis* homologs, the STRING network was generated and visualized at either the high confidence (.7) or highest confidence (.9) level.

## 3 RESULTS

### 3.1 Phenotypic results

For RNA‐seq analyses, leaf, stem, and root tissues from resistant plants were collected 12, 24, and 36 HAI with *P. gregata* or mock infection. As tissue was collected before the onset of BSR symptoms, additional seedlings of the resistant and susceptible genotypes (infected and mock infected) were allowed to grow for 5 wk to confirm expected BSR phenotypes. Disease screening was conducted using three standard methods: foliar severity, stem severity, and stem recovery of *P. gregata* (McCabe et al., 2016). Brown stem rot symptoms were not observed on resistant mock or susceptible mock plants, indicating they were healthy. Resistant infected plants appeared healthy with no BSR foliar symptoms and moderate stem browning. Susceptible infected plants had expected BSR foliar symptoms including necrosis and chlorosis of the leaves and also severe stem browning. Using all three screening methods, appropriate BSR phenotypes were observed for each genotype × treatment combination (Supplemental Table S1), providing confidence for gene expression analyses. Since we were interested in disease resistance mechanisms, only resistant infected and resistant mock plants were used for RNA‐seq.

### 3.2 Gene expression patterns in response to *Phialophora gregata* infection

RNA sequencing libraries for 46 samples (15 leaf, 16 stem, and 16 root samples) were sequenced and mapped to the soybean reference genome, Wm82.a2.v1 (Schmutz et al., 2010). The samples contained over 1.1 billion 100‐bp single‐end reads (324,219,965 in leaves; 395,090,774 in stems; and 404,664,803 in roots), 96.7% of these mapped to the reference genome.

Significant DEGs (FDR < .05) responding to *P. gregata* infection were identified by comparing infected with mock infected in each tissue and time point (Table 1). The analysis resulted in thousands of DEGs in each tissue and across time: 5,958 in leaves (Supplemental Table S2), 4,953 in stems (Supplemental Table S3), and 3,758 in roots (Supplemental Table S4). We compared DEGs between time points within tissues (Table 1). In leaves, 2,677 DEGs were identified 12 HAI, 3,917 were identified 24 HAI, and 81 were identified 36 HAI. Only seven DEGs were expressed at all three time points in leaves. In stems 221, 3, and 4,809 DEGs were identified at 12, 24, and 36 HAI, respectively. None of these were DE at all three time points in stems. In roots, 66, 122, and 3,606 DEGs were identified 12, 24, and 36 HAI, respectively. Only one gene was DE at all three time points in roots. These results suggest that *P. gregata* infection elicits rapid gene expression changes across time and within different tissues. Further, of the 12,397 unique DEGs found across all tissues and time points, only 1,695 (13.7%) were shared between any two tissues and only 146 (1.2%) were shared between all three tissues. This suggested that all three tissues respond very differently to *P. gregata* infection.

**TABLE 1:** Number of significant (FDR < .001) differentially expressed genes (DEGs) responding to *Phialophora gregata* treatment in leaf, stem, and root tissue. DEGs induced (+) and repressed (−) are specified

Treatment effect (infected and mock)	12 h	24 h	36 h
Leaves	1784+, 893−	2304+, 1613−	47+, 34−
Stems	142+, 79−	3+, 0−	3035+, 1774−
Roots	55+, 11−	110+, 12−	1496+, 2110−

To determine if differences observed between tissues were due to timing of the response or different defense mechanisms to *P. gregata* infection, we identified significantly overrepresented (corrected *P* < .05) GO biological process terms among induced and repressed genes within each tissue and time point. This allowed us to identify different pathways contributing to defense mechanisms in each tissue. We identified 156, 88, and 77 overrepresented GO terms in leaves, stems, and roots, respectively (Supplemental Table S5). Of the 156 GO terms from leaves, 58 (65.9% of stem GO terms) and 43 (55.8% of root GO terms) were shared with stems and roots, respectively. Seven GO terms were significantly overrepresented in at least one time point in all three tissues. While only 13.7% of DEGs were shared between any two tissues, more than 55% of GO terms identified in one tissue overlapped with another tissue. This suggests the differences in DEGs observed between tissues are likely the result of timing and not different resistant mechanisms.

**FIGURE 1:**
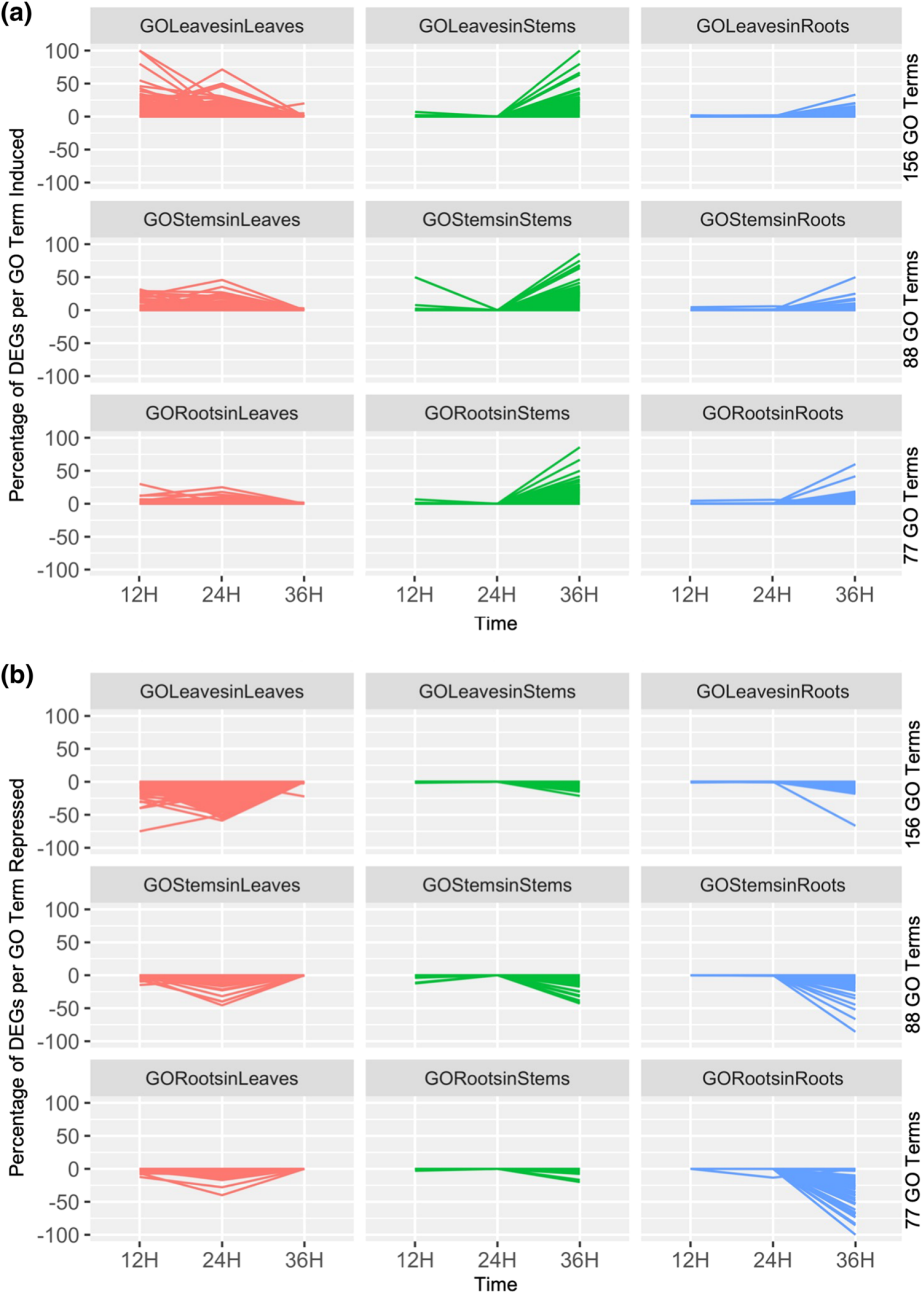
Leaves, stems, and roots use the same pathways to respond to *Phialophora gregata* infection. Significantly (corrected *P*‐value < .05) overrepresented biological process gene ontology (GO) terms were identified for each tissue by time point combination (Supplemental Table S5). There were 156, 88, and 76 GO terms identified in leaves, stems, and roots, respectively. Significant GO terms identified in each tissue were then used to query all three to tissues to identify gene expression patterns associated with each GO term (Supplemental Table S6). To give all GO terms equal representation regardless of GO term size, GO terms were plotted as a percentage of the number of differentially expressed genes (DEGs) in that GO term expressed in a particular tissue and time point relative to the total number of genes in the reference genome associated with that GO term. (a) Induced and (b) repressed genes were counted separately. This analysis reveals that all tissues respond to *P. gregata* infection using the same pathways; however, leaves respond first, well before stems and roots

To further investigate this, we examined how each GO term significantly overrepresented in one tissue responded in the other two tissues (Figure 1; Supplemental Table S6). For the 156 significant GO terms identified in the leaves, expression was detected 12 and 24 HAI in leaves but was not detected in stems and roots until 36 HAI. For the 88 and 76 significantly overrepresented GO terms from stem and roots, respectively, expression was largely detected only 36 HAI in stems and roots; however, expression was detected 12 and 24 HAI in the leaves. The same gene expression pattern was detected across GO terms identified from leaves, stems, and roots. This suggests that leaves, stems, and roots use the same pathways to respond to *P. gregata* infection; however, stems and roots are delayed in their response.

### 3.3 Genetic networks responding to *Phialophora gregata* treatment

**FIGURE 2:**
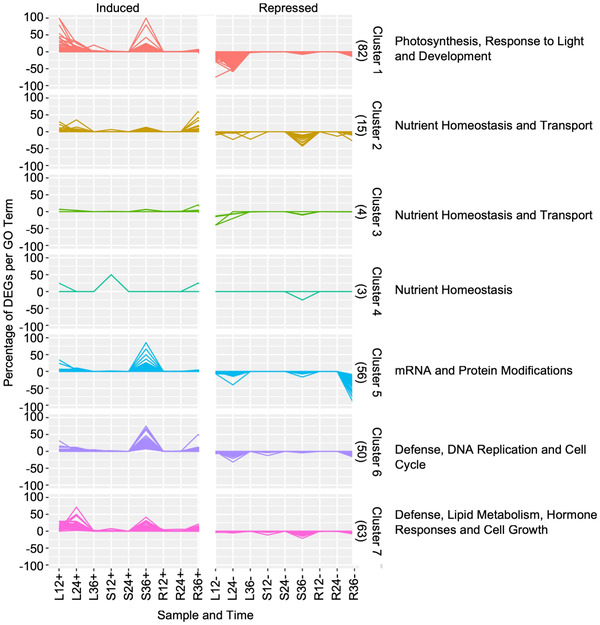
Clustering of gene ontology (GO) term data can be used to group pathways with similar function and expression patterns. We clustered gene expression patterns for the 273 unique GO terms identified in leaves, stems, and roots (Supplemental Table S6). To give all GO terms equal representation regardless of GO term size, GO terms were graphed as a percentage of the number of differentially expressed genes (DEGs) in that GO term expressed in a particular tissue and time point relative to the total number of genes in the reference genome associated with each GO term. Each GO term is unique to a cluster. This analysis grouped GO terms with similar expression patterns across tissues and points, resulting in seven clusters. The number of GO terms associated with each cluster is provided in parentheses beneath the cluster ID. In most cases, clustered GO terms had related biological functions. L, leaf; S, stem; R, root; +, induced; −, repressed; 12, 12 HAI; 24, 24 HAI; 36, 36 HAI

We clustered the 273 unique GO terms to compare gene expression patterns across tissues and time points. This resulted in seven GO clusters, each with unique expression signatures (Figure 2; Supplemental Tables S5 and S6). For example, Cluster 1 contained 82 GO terms; 61 of these GO terms were significant among repressed genes in leaves 12 and 24 HAI. These GO terms were largely associated with photosynthesis (electron transport, chloroplast organization, photosystem assembly, and repair), response to light (cellular response to light, response to blue, red and far red light), and development (leaf morphogenesis, ovule development and embryo development). Gene ontology terms involved in photosynthesis were unique to Cluster 1. Of the remaining GO terms in Cluster 1, 19 were significantly overrepresented among genes induced in stems 12 and 24 HAI. These GO terms were largely associated with general defenses (response to UV‐B, phenylpropanoid biosynthesis and metabolism, anthocyanin biosynthesis, defense response to bacteria) and growth and development (growth, regulation of asymmetric cell division and maintenance of floral organ identify). The gene expression signature of Cluster 2 (15 GO terms) included DEGs induced 36 HAI in roots and repressed 36 HAI in stems. Nine significant GO terms in Cluster 2 were largely associated with nutrient homeostasis and transport (phosphate ion homeostasis, phosphate ion transport, inorganic anion transport, cellular copper ion homeostasis, and zinc ion transmembrane transport). Nutrient homeostasis and transport were also significant in Cluster 3 (four GO terms) and Cluster 4 (three GO terms); however, significant GO terms were detected 12 HAI in repressed leaves (Cluster 3) and 12 HAI in induced stems (Cluster 4). Cluster 5 (56 GO terms), Cluster 6 (50 GO terms), and Cluster 7 (63 GO terms) each had induced gene expression peaks 36 HAI in stems. However, the clusters could be distinguished by the tissue in which GO terms were significant. Of the 56 GO terms in Cluster 5, 52 were significantly overrepresented in roots 36 HAI. Of the 50 significant GO terms in Cluster 6, 36, and 10 were significantly induced in stems and roots 36 HAI, respectively. Of the 63 GO terms in Cluster 7, 51 were significant among *P. gregata*‐induced DEGs in leaves 12 and 24 HAI. Cluster 5 contained GO terms associated with mRNA and protein modifications (RNA methylation, mRNA splicing and maturation, rRNA and mRNA export, protein folding, response to misfolded proteins, protein targeting, protein maturation, and protein import). Cluster 6 contained GO terms associated with defense (response to fungus, jasmonic acid (JA) metabolic process, response to chitin, salicylic acid (SA)‐mediated signaling, respiratory burst involved in defense, hypersensitive response, and systemic acquire resistance), and DNA replication and cell cycle (nuclear division, spindle assembly, RNA interference, DNA methylation, chromatin silencing, and regulation of cell cycle). Cluster 7 was associated with general defense responses (response to nematode, salt stress, karrikin, wounding, and JA), lipid metabolism (lignin biosynthetic process, lipid biosynthesis, and very long chain fatty acid biosynthesis), hormone responses (response to auxin, ethylene, regulation of hormone levels, and JA biosynthesis), and cell growth (floral whorl, xylem and meristem development, flower morphogenesis, cell tip growth, and root hair elongation). For the majority of clusters (Clusters 1, 2, 5–7), there are definable peaks and valleys corresponding to each tissue (leaves, stems, roots), suggesting the tissues are using the same pathways at different times but with different gene repertoires.

The surprising speed of the defense response and dynamic gene expression changes across time support the involvement of TFs in regulating defense responses to *P. gregata* infection. We used the SoyDB TF database (Wang et al., 2010) to identify DE TFs within each tissue and time point (Supplemental Table S7). In response to *P. gregata* treatment, we identified 684 TFs in leaves (217 at 12 HAI, 453 at 24 HAI, and 14 at 36 HAI), 641 TFs in stems (14 at 12 HAI, 0 at 24 HAI, and 627 at 36 HAI), and 324 TFs in roots (six at 12 HAI, 13 at 24 HAI, and 305 at 36 HAI). Of the total 1,299 TFs identified across tissues, 409, 406, and 225 TFs were unique to leaves, stems, or roots, respectively. These TFs represent 57 different transcription factor families (TFFs). Of 57 TFFs identified, 47 TFFs (82%) were shared between two or more tissues at any time point. This again suggests timing differences on an individual gene scale but shared response networks between tissues.

**FIGURE 3:**
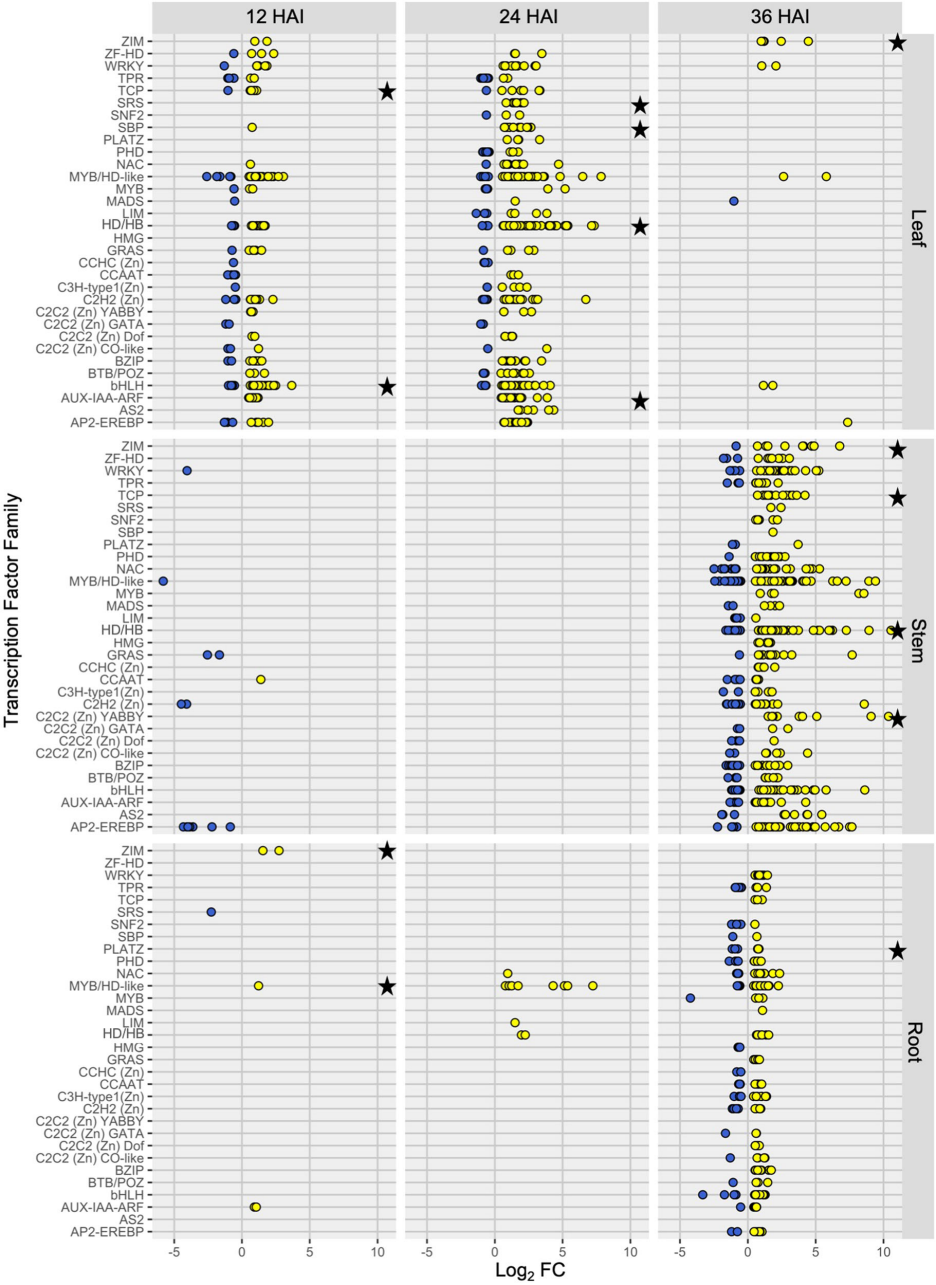
Transcription factor (TF) expression in response to *Phialophora gregata* infection in leaves, stems, and roots across time. Significantly (false discovery rate <.05) differentially expressed TFs were identified in each tissue by time point combination. Log‐2 fold change is plotted on the *x*‐axis and TF families are plotted on the *y*‐axis. For visualization purposes, log‐2 fold changes were limited to ±10. Transcription factors families with <10 significant differentially expressed genes (DEGs) were removed. For the complete data set, see Supplemental Table S6. Stars indicate significantly (corrected *P* < .05) overrepresented TF families

In each tissue and time point, we graphed TF expression by TFF (Figure 3; Supplemental Table S7) noting several trends. First, regardless of tissue, the majority of TFs are induced. Second, TF expression in leaves starts 12 HAI infection; however, the majority of TF expression in roots and stems is not detected until 36 HAI. Third, the strength of TF expression is much higher in leaves and stems than roots.

While looking at individual TFF expression can provide important biological insights, it is worth noting that the size of TFFs can vary significantly. To adjust for this, we also identified TFFs that were significantly overrepresented within a specific tissue and time point relative to their genome representation. Of the 57 TFFs, we identified 10 TFFs that were significantly overrepresented (Corrected *P* < .05). In leaves, basic helix‐loop‐helix and TCP TFFs were significantly overrepresented 12 HAI; AUX‐IAA‐ARF, Homeodomain/HOMEOBOX, SBP, and SRS TFFs were significantly overrepresented 24 HAI; and the ZIM TFF was significantly overrepresented 36 HAI. In stems, TCP, Homeodomain/HOMEOBOX, ZIM, and C2C2 (Zn) YABBY TFFs were significantly overrepresented 36 HAI. In roots, ZIM and MYB/HD‐like TFFs were significantly overrepresented 12 HAI, and the PLATZ TFF was significantly overrepresented 36 HAI.

## 4 DISCUSSION

Brown stem rot is a major soybean disease in the United States; fields infected with *P. gregata* can experience yield losses of up to 38% (Bachman & Nickell, 2000). Brown stem rot symptoms do not appear until 5 WAI, and phenotyping requires multiple destructive measurements. This delays resistance breeding and screening efforts. Therefore, understanding and characterizing the resistance response to *P. gregata* is of great economic importance. Identifying signatures of the BSR defense responses immediately after infection will allow us to develop molecular tools to streamline the screening process.

### 4.1 Evidence for biphasic defense signaling in soybean–*Phialophora gregata* interactions

A previous BSR expression study (McCabe et al., 2018) identified genes responding to *P. gregata* 1 WAI. In that study, a large response was observed in leaf tissue of the resistant genotype 7 DAI (2,492 total DEGs); however, little differential gene expression was observed in roots (16 total DEGs) and stems (530 DEGs). Further, little overlap in gene expression was seen between resistant and susceptible lines in response to infection. Since this initial experiment focused on responses 7 DAI with *P. gregata*, we hypothesized that the early defense signaling occurring in stems and roots had already passed.

Therefore, this study analyzed genes DE in response to *P. gregata* infection or mock infection at 12, 24, and 36 h in resistant leaves, stems, and roots. These time points also coincide with germination of *P. gregata* conidia (Allington & Chamberlain, 1948) and the movement of fungal spores through the stem vascular system reported by (Tabor et al., 2007). We identified thousands of genes responding to *P. gregata* infection in each tissue. Surprisingly, leaves, stems, and roots responded to *P. gregata* infection at different times, with leaves appearing to respond fastest. In Figures 1 and 2, we demonstrate that the same pathways are responding to *P. gregata* infection in all three tissues. Although *P. gregata* inoculation occurred in the stem, the majority of differential gene expression 12 HAI was seen in leaves. We believe differences in gene expression patterns in leaves, stems, and roots are likely associated with biphasic defense responses associated with PTI and ETI.

In soybean, biphasic defense responses have been best characterized in resistance to the fungal pathogen *Phakopsora pachyrhizi*, the cause of Asian soybean rust (Meyer et al., 2009; Morales et al., 2013; Schneider et al., 2011; van de Mortel et al., 2007). Microarray analyses revealed that resistant (carrying *Rpp2*, *Rpp3*, or *Rpp4*) and susceptible genotypes both responded to *P. pachyrhizi* 12 HAI, likely through recognition of PAMPs or damage‐associated molecular patterns (Choi & Klessig, 2016) leading to PTI. However, at 24 HAI little differential gene expression was observed, likely as a result of suppression of defense responses by the pathogen. By 72 HAI, only resistant genotypes initiated a second, more robust defense response. In this case, pathogen effectors were directly or indirectly recognized by plant resistance genes (*R* genes), leading to ETI. Interestingly, the biphasic responses observed were not limited to defense‐associated genes but also included genes associated with stress, metabolism, photosynthesis, transcription, and transport. While *P. pachyrhizi* is considered an obligate biotroph, it acts as a necrotroph in the early stages of infection, directly penetrating epidermal cells between 12 to 24 HAI. Between 72 and 96 HAI, the epidermal cells become necrotic and die while fungal growth continues (Campe, Loehrer, Conrath, & Goellner, 2014; Loehrer, Langenbach, Goellner, Conrath, & Schaffrath, 2008; Schneider et al., 2011).

Gene ontology terms within GO Clusters 1 and 7 were associated with the earliest and strongest differential gene expression in leaf tissue. Differential gene expression in leaves was detected at 12 and 24 HAI but disappeared by 36 HAI (Figure 2). Comparing DEGs from resistant leaves in this study (12, 24, and 36 HAI) with resistant leaves 7 DAI from our previous study (McCabe et al., 2018) identified 700 DEGs in common. Taken together, this suggests a potential biphasic defense response, where differential gene expression occurred 12 and 24 HAI in the leaves but was suppressed 36 HAI. The second wave of gene expression was captured in our previous experiment 7 DAI. Therefore, evidence of PTI might be present in the 12 and 24 HAI leaf data. The majority of GO terms associated with Cluster 1 (61 of 82; Supplemental Table S6) were involved in photosynthesis, response to light, and development. Gene ontology terms associated with photosynthesis, electron transport, chlorophyll biosynthesis, and response to light were repressed in leaves 12 and 24 HAI (Supplemental Table S6) as has been reported for resistant soybean responses to *P. pachyrhizi* (Morales et al., 2013; Schneider et al., 2011; van de Mortel et al., 2007).

Cluster 1 also contained a single‐defense GO term, incompatible defense response to bacteria (GO:0009816), containing 22 genes significantly induced 12 HAI. Of these genes, 17 were homologs of *AtLOX1* while four were single genes homologous to *AtRIN4*, *AtRPS5*, *AtACD1*, and *AtPLDBETA*. Each of these genes are associated with plant defense mechanisms in *Arabidopsis*. *AtPLDBETA* is induced 30–60 min after infection with *Pseudomonas syringae* likely through PTI (de Torres Zabela, Fernandez‐Delmond, Niittyla, Sanchez, & Grant, 2002). *AtLOX1* gene expression peaks 12 HAI with *P. syringae* (Melan et al., 1993). *AtRPS5* is a nucleotide‐binding site–leucine‐rich repeat (NBS‐LRR) resistance gene that confers ETI to multiple bacterial pathogens and downy mildew (Warren, Henk, Mowery, Holub, & Innes, 1998). *AtRIN4* is a negative regulator of PAMP‐induced gene signaling (Afzal, da Cunha, & Mackey, 2011). Cluster 7 (Supplemental Table S6) contained 15 GO terms significantly induced 12 HAI in the leaves that were associated with secondary metabolism and the production of phytoalexins, antimicrobial compounds, and cell‐wall reinforcing metabolites, all important components of general defense responses. Gene ontology terms associated with general defense were similarly induced in response to *P. pachyrhizi* infection during the first phase of infection of both resistant and susceptible soybean cultivars. However, in the second phase of infection, expression occurred earlier and stronger in resistant interactions (Morales et al., 2013; Schneider et al., 2011; van de Mortel et al., 2007).

The differential expression of these genes suggest the defense responses observed in the leaves 12 and 24 HAI with *P. gregata* likely are due to systemic spread of defense signaling from the site of infection in the stem to the leaves. In plants, long‐distance signaling to distant tissues can occur though long‐range transport of RNA or proteins, vascular hydraulic signals, hormones and small signaling molecules, electrical signaling, and long‐distance reactive oxygen species signaling (Choi, Hilleary, Swanson, Kim, & Gilroy, 2016). These signals can operate at speeds up to 105.5 m/s^−1^. Long‐distance signaling to distant tissues has been reported for both abiotic and biotic stress (Lai et al., 2018; Li et al., 2017; Wang et al., 2019). Recently, Wang et al. (2019) reported that infection by the root‐knot nematode (*Meloidogyne incoginita*) in tomato (*Solanum lycopersicum* L.) resulted in systemic defense signaling via electrical and reactive oxygen species, leading to increased JA synthesis and activation of mitogen‐activated protein kinases in the leaves. Further, nematode infection induced changes in the surface potential of stem, petioles, and leaf lamina and the cytoplasmic potential of leaf cells minutes to hours after infection.

### 4.2 The defense and growth trade off

In a one‐of‐a‐kind study, Tian, Traw, Chen, Kreitman, and Bergelson (2003) demonstrated that *Arabidopsis* plants carrying the *P. syringae* resistance gene *RPM1* had 9% fewer seeds per plant than identical plants missing *RPM1* when grown in a pathogen‐free environment, suggesting pathogen detection comes at a cost. Silencing either *MITOGEN‐ACTIVATED PROTEIN KINASE 4* (*GmMPK4*, [Liu et al., 2011]) or *MAPK KINASE KINASE1* (*GmMEKK1*, [Xu et al., 2018]) in soybean in the absence of a pathogen resulted in a strong hypersensitive response and localized cell death. At the same time, genes involved metabolism, photosynthesis, growth, and development were repressed. Similar trends were observed in resistance responses to *P. pachyrhizi* (Morales et al., 2013; Schneider et al., 2011), *P. syringae* (Zou et al., 2005), and soybean mosaic virus (SMV) (Babu, Gagarinova, Brandle, & Wang, 2008), representing soybean responses to fungal, bacterial, and viral pathogens. Bilgin et al. (2010) demonstrated the repression of photosynthesis‐related genes in response to biotic pathogens occurred in eight different plant species in response to a broad range of pathogens. Resistance responses are associated with increased demands for energy resulting in a fitness cost to the plant (Bolton, 2009). By downregulating energy‐expensive processes like cell growth and photosynthesis, resources can be diverted and used for defense responses and defense signaling networks. In each of the cases described above, expression studies were restricted to leaves and aboveground tissues. In this study, inhibition of photosynthesis was restricted to the leaves; however, repression of growth and development could be detected in leaves, stems, and roots (Supplemental Table S5), demonstrating that resources throughout the plant are redirected to mount sustainable pathogen defense responses.

### 4.3 Nutrient homeostasis

**FIGURE 4:**
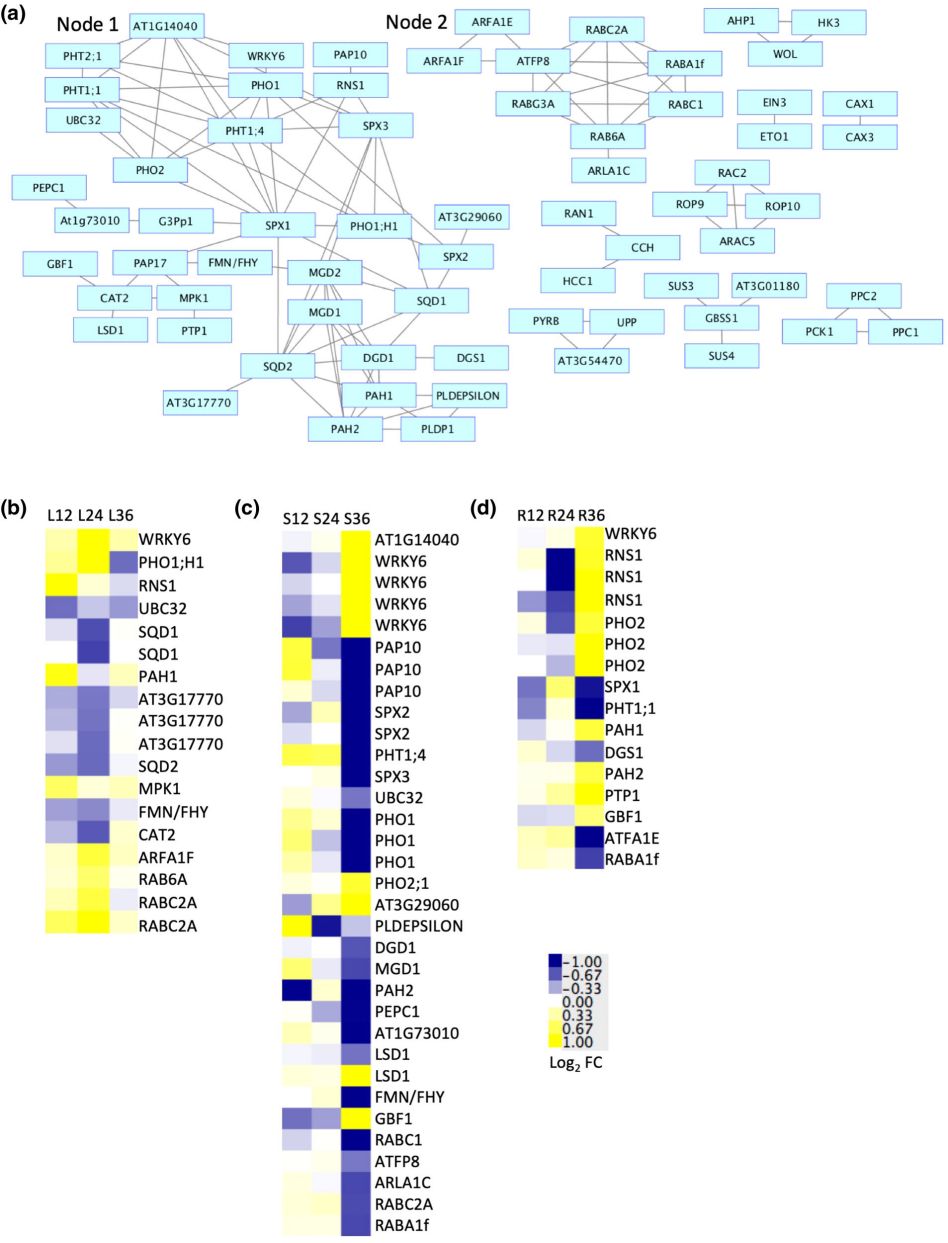
Clustered gene ontoloty (GO) terms associated with nutrient homeostasis form a phosphate homeostasis and defense network. The 15 GO terms from GO Cluster 2 (Figure 2; Supplemental Table S6) corresponded to 280 unique soybean genes homologous to 144 unique *Arabidopsis* proteins. To understand how these proteins interact, we used the STRING network (version 11), limiting network associations to those with a high confidence score (confidence > .70; Supplemental Table S8). The (a) resulting network contained 67 *Arabidopsis* proteins grouped into networks of three or more genes. Expression patterns of soybean genes corresponding to Nodes 1 and 2 were analyzed. Expression of genes unique to (b) leaves, (c) stems, or (d) roots are shown. Expression patterns were limited to gene uniquely differentially expressed in a tissue to aid in visualization

Foliar BSR symptoms can be confused with foliar symptoms of soybean sudden death syndrome, stem canker, and other diseases (Crop Protection Network, 2019; Penn State University Extension, 2018; University of Nebraska‐Lincoln Cropwatch, 2015). In addition, foliar BSR disease symptoms can be confused with nutrient deficiencies (IlliniFS, 2019; Montana State University of Extension, 2011), both causing interveinal chlorosis of the leaves, stunting, and reduced yield in susceptible genotypes. With this in mind, Cluster 2 of our GO analysis stood out because of its association with nutrient acquisition. Of the 15 GO terms in Cluster 2, seven were associated with nutrients including phosphate (cellular response to phosphate starvation [GO:0016036], phosphate ion transport [GO:0006817], and phosphate ion homeostasis [GO:0055062]), copper (cellular cooper ion homeostasis [GO:0006878]), sulfur (sulfate transport [GO:0008272]), manganese (cellular manganese ion homeostasis [GO:0030026]), and zinc (zinc ion transmembrane transport [GO:0071577]). The 15 GO terms corresponded to 280 unique soybean genes homologous to 144 unique *Arabidopsis* proteins (Supplemental Table S8). To understand how these proteins interact, we used the STRING network (Szklarczyk et al., 2018), limiting the output to networks meeting a high confidence score (confidence >.70). The resulting network contained 67 *Arabidopsis* proteins grouped into networks of three or more genes. Of particular interest were two networks (Node 1 and Node 2) containing 36 and nine proteins, respectively (Figure 4a).

Node 1 contained proteins associated with phosphate transfer, phosphate scavenging and induction of phosphate starvation induced genes, and proteins associated with defense and cell death. AtPHO1, AtPHO1;H1, AtPHO1;H3 (AT1G14040), AtPHO2, AtPHT1;1, AtPHT1;4, AtPHT2;1, EXS (AT3G29060,) and AtWRKY6 are involved in phosphate transport and regulation of phosphate starvation responses throughout the plant (Chen et al., 2009; Khan et al., 2014; Liu et al., 2012; Secco et al., 2017; Versaw & Harrison, 2002). Under phosphate sufficiency, AtSPX proteins bind AtPHR1 (not DE) to prevent it from binding to promoters of phosphate starvation induced genes (Puga et al., 2014). AtRNS1, AtPS2 (At1G73010), AtPEPC1, AtPAH1, AtPAH2, AtSQD1, AtSQD2, AtMGD1, AtMGD2, AtDGD1, AtDGS1, and AtPLPEPSILON are involved in scavenging phosphate from phospholipids to produce galactolipids, sulfolipids, and free phosphate (Bariola et al., 1994; Nakamura et al., 2009; Thibaud et al., 2010). The majority of genes involved in phosphate uptake and remobilization are repressed in stems 36 HAI (Figure 4c), suggesting that inhibiting the uptake and remobilization of phosphate is important to defense. As a necrotroph, *P. gregata* might acquire nutrients such as phosphate from the plant. Limiting free phosphate may then inhibit *P. gregata* growth and establishment. Impullitti and Malvick (2014) found that in the susceptible soybean line Corsoy 79, *P. gregata* Type A could infect xylem vessels. In contrast, the resistant line Bell limited infection to the interfascicular regions of the stem, blocking access to nutrients and water found in the xylem. This hypothesis is supported by the recent publication by Dong et al. (2018) that identified the differential expression of genes and GO categories related to ion homeostasis in the resistant response to soybean downy mildew (*Peronospora manshurica)*, suggesting that modification of nutrient availability plays a vital role in the defense response.

Node 1 also contained a number of defense‐ and senesce‐related proteins including AtCAT2, AtLSD1, AtGBF1, AtMPK1, and AtPTP1. AtCAT2 regulates SA‐mediated repression of JA biosynthesis during defense (Yuan, Liu, & Lu, 2017). AtGBF1 and AtLSD1 interact with AtCAT2 to regulate hypersensitive cell death (Giri et al., 2017; Li, Chen, Mu, & Zuo, 2013). AtPTP1 dephosphorylates members of mitogen‐activated protein kinase (MAPK) cascades to repress SA biosynthesis and regulate defense responses (Bartels et al., 2009). Node 2 contained three ARF GTPases and six Rab GTPases. In general, the GTPase superfamily is involved in endomembrane trafficking (for complete review see Rivero, Traubenik, Zanetti, & Blanco, 2017). During resistance signaling, GTPases traffic proteins important in pathogen defense such as receptors and antimicrobial compounds. Further, GTPases can be targeted by pathogen effectors to perturb the delivery of defense compounds to the cell surface. The presence of defense and phosphate homeostasis genes in the same network provide support that regulating phosphate reserves is an intrinsic defense response to *P. gregata*.

### 4.4 Characterizing defense responses to *Phialophora gregata*

**FIGURE 5:**
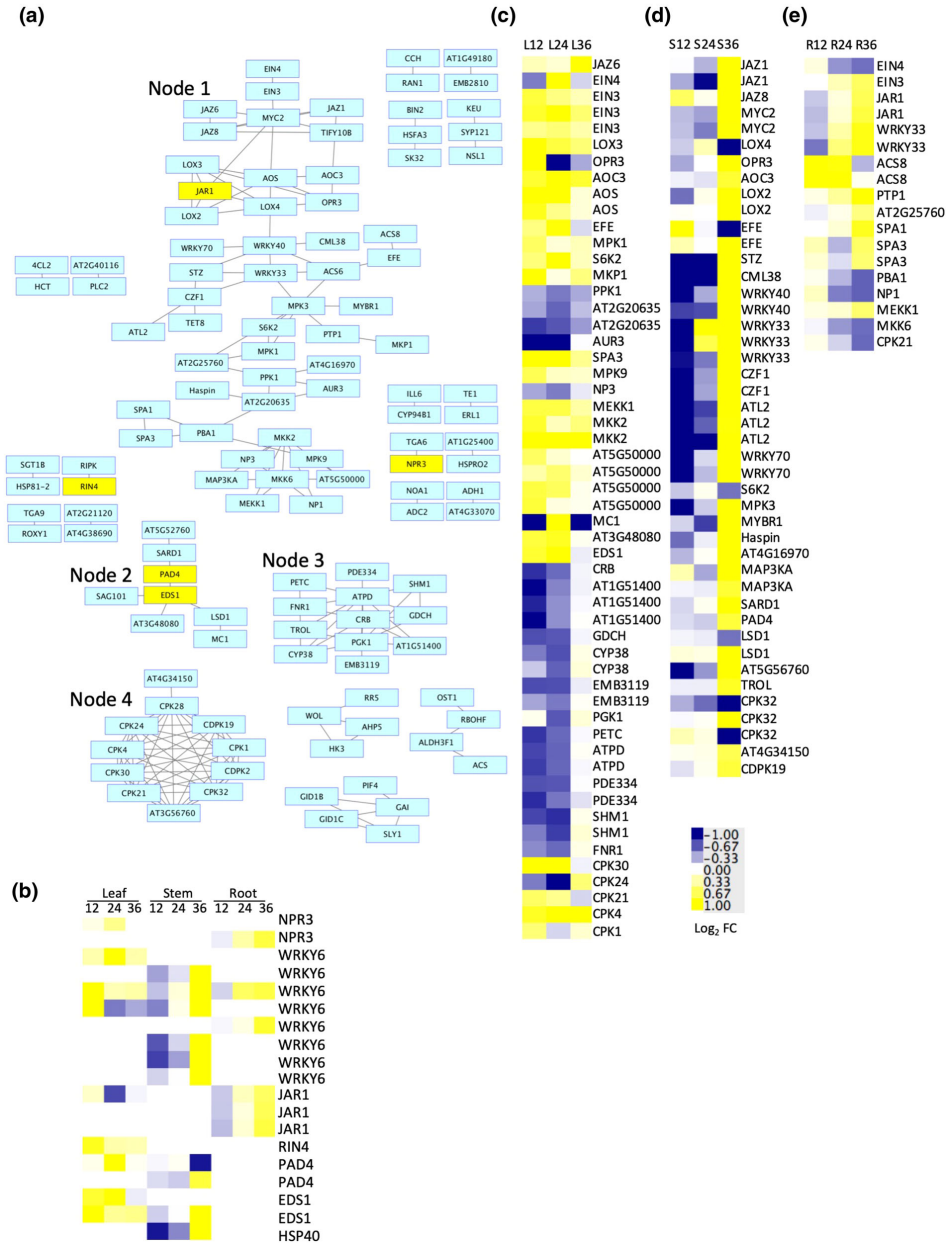
Clustered gene ontology (GO) terms associated with defense responses form a defense network. The 13 GO terms associated with defense in GO Cluster 6 (Figure 2; Supplemental Table S6) corresponded to 1,170 unique soybean genes homologous to 621 unique *Arabidopsis* proteins. To understand how these proteins interact, we used the STRING network (version 11), limiting network associations to those with the highest confidence score (confidence >.90; Supplemental Table S9). (a) The resulting network contained 126 *Arabidopsis* proteins grouped into nodes of three or more genes. (b) Soybean genes required for resistance to different pathogens are highlighted yellow, their expression across tissues, and time points is shown in panel. Expression patterns of soybean genes corresponding to Nodes 1 through 4 were analyzed. Expression of genes unique to (c) leaves, (d) stems, or (e) roots are shown

To identify the genes and networks contributing to ETI to *P. gregata*, we focused our analysis on GO Cluster 6 with strong differential expression in stems or roots 36 HAI. Of the 50 GO terms in Cluster 6, 13 were associated with defense (respiratory burst involved in defense [GO:0002679], response to fungus [GO:0009620], systemic acquired resistance [GO:0009862], SA‐mediated signaling [GO:0009863], response to chitin [GO:0010200], regulation of plant hypersensitive response [GO:0010363], negative regulation of defense response [GO:0031348], JA metabolism [GO:0009694], and SA biosynthesis [GO:0009697]), stress (response to ozone [GO:0010193] and response to mechanical stimulus [GO:0009612]) or signaling (protein phosphorylation [GO:0006468] and intracellular signal transduction [GO:0035556]). These 13 defense GO terms corresponded to 1,170 unique soybean genes, which were homologous to 621 unique *Arabidopsis* proteins (Supplemental Table S9). To allow visualization of the data, we created a network using STRING (Szklarczyk et al., 2018) and limited the output to networks meeting the most stringent interaction confidence score (confidence >.90). The resulting network contained 126 *Arabidopsis* proteins. We identified four networks (Nodes 1–4; Figure 5a–5e), the majority of which are induced 36 HAI with *P. gregata* in the stem (Figure 5d).

Node 1 contained 48 proteins largely associated with plant defense. AtAOS, AtAOC3, AtOPR3, AtJAR1, AtLOX2, AtLOX3, and AtLOX4 are all involved in the biosynthesis of biologically active Jasmonyl‐L‐Isoleucine (Chauvin, Caldelari, Wolfender, & Farmer, 2013; Wasternack & Strnad, 2019). Jasmonyl‐L‐Isoleucine signaling during pathogen attack leads to the degradation of JAZ and TIFY proteins (AtJAZ1, AtJAZ6, AtJAZ8, and AtTIFY10B), releasing suppression of the AtMYC2 TF (Kazan & Manners, 2013). AtMYC2 forms homodimers and heterodimers with other MYC TFs to promote transcription of JA‐responsive genes and inhibit transcription of ethylene‐responsive genes (AtEIN3 and AtEIN4; [Zhang et al., 2014]). This node also contains a number of important TFs such as AtWRKY33, AtWRKY40, AtWRKY70, AtSTZ, and AtCZF1. AtWRKY33 and ATWRKY40 bind to the promoters of genes involved in JA and SA signaling (Birkenbihl, Kracher, Roccaro, & Somssich, 2017). AtWRKY70 inhibits immune responses in the absence of pathogens but activates immune responses during pathogen attack (Zhou et al., 2018). Both AtSTZ and AtCZF1 are involved in abiotic stress signaling (Park et al., 2015). Similarly, MAPK modules (consisting of a MAPK, a MAPK Kinase [MAP2K] and a MAPKK Kinase [MAP3K]) regulate proteins through phosphorylation cascades (Colcombet & Krysan, 2018). AtMAP3KA, AtMPK3, AtMKK6, and AtMEKK1 are involved in PTI (Colcombet & Krysan, 2018; Wersch, Gao, & Zhang, 2018). AtMPK3 is also involved in ETI. AtMKK2 and ATMPK1 are involved in abiotic stress signaling (Boudsocq, Danquah, de Zélicourt, Hirt, & Colcombet, 2015; Teige et al., 2004).

Node 2 contained eight proteins including AtEDS1, AtPAD4, AtSARD1, AtSAG101, AtLSD1, and AtMC1. AtEDS1 forms heterodimers with AtPAD4 and AtSAG101 to regulate ETI (Bhandari et al., 2019). An AtEDS1–AtPAD4–AtSAG101 complex is required for resistance against turnip crinkle virus (Zhu et al., 2011). The AtEDS1–AtPAD4 heterodimer and AtSARD1 regulate expression of *AtICS1* involved in SA biosynthesis (Cui et al., 2018; Zhang et al., 2010). Both AtPAD4 and AtSAG101 are required for programmed cell death regulated by NBS‐LRR *R*‐genes (Feys et al., 2005). While AtMC1 is a positive regulator of programmed cell death (Coll et al., 2014), AtLDS1 is negative regulator (Li et al., 2013).

Nodes 1 and 2 exemplify the paradox between SA and JA signaling. Salicylic acid regulates the hypersensitive response and programmed cell death, which benefit necrotrophic pathogens such as *P. gregata* by providing readily assessable nutrients (see review by Lorang et al., 2019). Therefore, suppression of SA signaling and induction of JA‐mediated signaling is essential to defense against necrotrophs. In Nodes 1 and 2, we can see that *P. gregata* infection is inducing the biosynthesis of JA and positively regulating JA‐mediated defense genes. Interestingly, resistance to necrotrophic pathogens is often associated with susceptibility genes and not *R*‐gene‐mediated resistance. However, we know BSR resistance has been mapped to a region of the soybean genome containing multiple clusters of receptor‐like proteins (McCabe et al., 2018). This suggests that receptor‐like proteins in this region recognize *P. gregata* effectors and initiate JA signaling to defend against attack.

Node 3 contained 12 photosynthesis related proteins including At1G51400, AtCRB, AtCYP38, AtEMB3119, AtFNR1, AtGDCH, AtPDE334, AtSHM1, and AtTROL. Both the pigment defective (AtPDE334) and embryo defective (AtEMB3119) proteins are localized in the chloroplast (Bryant, Lloyd, Sweeney, Myouga, & Meinke, 2011) and AtCRB has a role in regulating transcripts in the chloroplast (Hassidim et al., 2007). Chloroplasts produce the oxidative burst leading to a hypersensitive‐response‐type programmed cell death. AtGDCH and AtSHM1 are involved in photorespiration and leaf senescence (Voll et al., 2006; Zhou et al., 2013). AtCYP38, AtTROL, AtFNR1, and At1G51400 play roles in the assembly, maintenance, and efficiency of energy generation in Photosystem 2 (Sirpiö et al., 2008; Vojta et al., 2015) as the demand to convert light energy into chemical energy is increased when energy‐costly defense responses are deployed (Göhre, Jones, Sklenář, Robatzek, & Weber, 2012).

Node 4 contained nine calcium‐dependent protein kinases (AtCPK1, AtCDPK2, AtCPK4, AtCPK19, AtCPK21, AtCPK24, AtCPK28, AtCPK30, and AtCPK32), one calcium‐dependent lipid binding protein, and one protein kinase. Calcium‐dependent protein kinases are involved in plant development and abiotic and biotic stress responses (Gao et al., 2013; Shi et al., 2018). Calcium‐dependent protein kinases interpret calcium signals generated by various stimuli to rapidly initiate signaling cascades. AtCPK1 controls the onset of localized cell death and production of reactive oxygen species in *RPS2‐* and *RPM1*‐mediated defense responses to *P. syringae* (Gao et al., 2013). AtCPK4 and AtCPK28 regulate levels of defense hormones ethylene and JA (Shi et al., 2018).

Remarkably, several of the soybean genes identified in the GO Cluster 6 network are required for different *R*‐gene‐mediated signaling networks in soybean. GmNPR1 (Glyma.09G020800, corresponds to AtNPR3 in Figure 5) and GmWRKY36 (Glyma.12G097100 and Glyma.13G310100, corresponds to WRKY6, Supplemental Table S9) are required for *Rpp2*‐mediated resistance to *P. pachyrhizi* (Pandey et al., 2011). GmJAR1 (Glyma.07G057900, Node 1; Figure 5) is required for *Rsv1*‐mediated resistance to SMV (Zhang et al., 2012). GmRIN4 (Glyma.16G090700) is required for *Rpg1‐b‐*mediated resistance to *P. syringae* (Selote et al., 2013). GmPAD4 (Glyma.13G069800, Node 2; Figure 4) is required for both *Rpp2‐* and *Rsv1‐* mediated signaling (Pandey et al., 2011; Zhang et al., 2012). GmEDS1 (Glyma.04G177700 and Glyma.06G187400, Node 2; Figure 5) is required for *Rpp2‐, Rsv1‐*, and *Rpg2*‐mediated signaling to a fungal, viral, and bacterial pathogen, respectively (Pandey et al., 2011; Wang et al., 2014; Zhang et al., 2012). Silencing of *GmHSP40* (Glyma.13G257100 and Glyma.15G057800; Supplemental Table S9) enhanced susceptibility to SMV (Liu & Whitham, 2013). It is important to highlight that soybean proteins in this network are required for resistance to a broad spectrum of pathogens including viruses, fungi, and bacteria. Further, proteins such as EDS1 and PAD4 also have roles in abiotic stress tolerance and plant productivity such as biomass production, yield, photosynthetic efficiency, and water use efficiency in other species (Bernacki, Czarnocka, Szechyńska‐Hebda, Mittler, & Karpiński, 2019). The fact that seven genes in this cluster are required for resistance to soybean pathogens suggests this cluster represents a central hub of defense proteins, many whose function has not yet been characterized. It is likely that novel proteins in this cluster will prove to be equally important in defense against pathogens and may enhance plant productivity. Therefore, these genes are obvious targets for future crop improvement.

In summary, resistance to *P. gregata* has been difficult to find and manage, requiring intense, disruptive phenotyping assays. Unlike most pathogens, *P. gregata* is a very slow growing necrotroph that infects the plants in two stages. Following infection, a latent phase lasts for several weeks with no visible disease symptoms in resistant or susceptible lines. This is followed by a pathogenic phase where disease symptoms begin to develop in both resistant and susceptible lines (Impullitti & Malvick, 2014). Our study demonstrates that a resistant soybean line recognizes *P. gregata* infection within 12 h, leading to PTI. By 36 h, *R*‐gene‐mediated defenses promote ETI using JA signaling. Future research will compare resistance responses across diverse resistant germplasm to identify resistance signatures associated with the three known *Rbs* resistance genes. In addition, we will incorporate virus‐induced gene silencing to knock down central genes in the defense cluster network. This will allow us to exploit the gene networks described here to develop novel diagnostic tools, enabling faster identification of novel resistant germplasm and integration of resistance into elite germplasm.

## AUTHOR CONTRIBUTIONS

The authors each planned and designed the research, performed the experiment, analyzed the data, and wrote the manuscript.

## CONFLICT OF INTEREST

The authors declare that they have no competing interests.

## Supporting information

Supplementary Material

## Data Availability

The RNA‐seq data generated and analyzed in the current study are available at the National Center for Biotechnology (NCBI) Short Read Archive (SRA) Bioproject accession PRJNA548564. Additional datasets supporting the conclusions of this article are included within the article and the supplementary tables.
